# Supplementary material for: Retrospective spatial analysis for African swine fever in endemic areas to assess interactions between susceptible host populations
Source: PLoS One. 2020 May 29;15(5):e0233473. doi: 10.1371/journal.pone.0233473 (PMC7259610; doi:10.1371/journal.pone.0233473)
Supplement: S1 Appendix — For definitions of variables see section 2.3 (material and methods). (DOCX) [file pone.0233473.s001.docx]

Supplementary Material

**S1 Appendix**


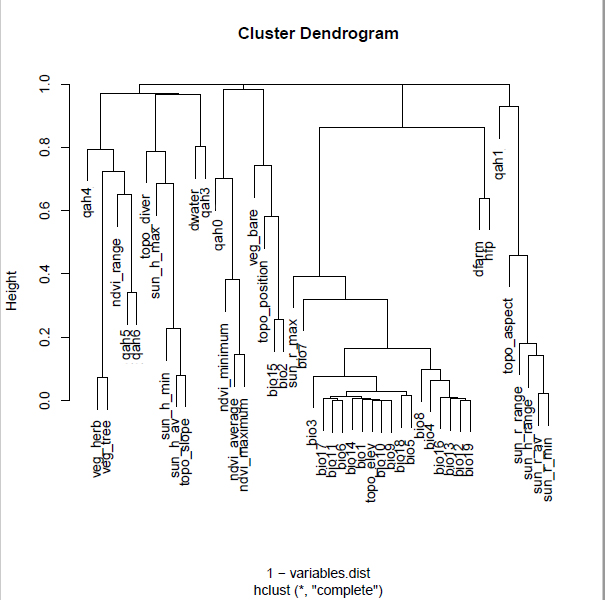


**S1 Appendix.** Cluster dendrogram or correlation matrix of the environmental variables included as predictors in the latent selection difference function (LSD) approach. For definitions of variables see section 2.3 (material and methods).
