# Supplementary material for: Retrospective spatial analysis for African swine fever in endemic areas to assess interactions between susceptible host populations
Source: PLoS One. 2020 May 29;15(5):e0233473. doi: 10.1371/journal.pone.0233473 (PMC7259610; doi:10.1371/journal.pone.0233473)
Supplement: S3 Appendix — Average distance (in meters) per hour (a) and average distance (meters) per month (b) for wild boar number 7000 (adult male), 7001 (adult male) and 7003 (adult female) for the period from March 2014 to September 2014. (DOCX) [file pone.0233473.s003.docx]

**S3 Appendix**


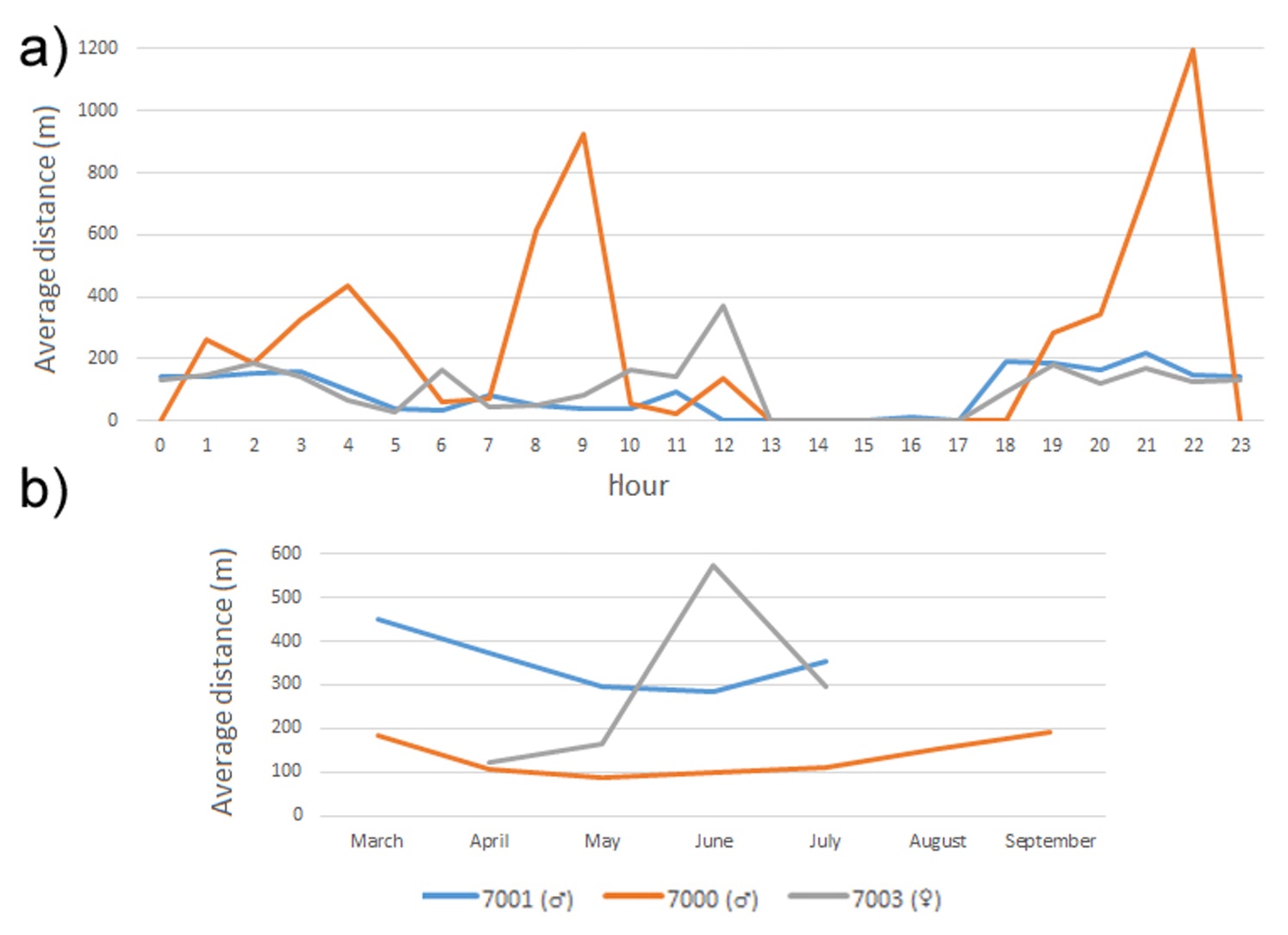


**S3 Appendix.** Average distance (in meters) per hour (a) and average distance (meters) per month (b) for wild boar number 7000 (adult male), 7001 (adult male) and 7003 (adult female) for the period from March 2014 to September 2014.
