# Supplementary material for: Retrospective spatial analysis for African swine fever in endemic areas to assess interactions between susceptible host populations
Source: PLoS One. 2020 May 29;15(5):e0233473. doi: 10.1371/journal.pone.0233473 (PMC7259610; doi:10.1371/journal.pone.0233473)
Supplement: S4 Appendix — The observed frequencies of free-ranging pig locations in the validation dataset are plotted as a function of the predicted probability that the habitat would be used by free-ranging pigs in relation to wild boar. (DOCX) [file pone.0233473.s004.docx]

S4 Appendix


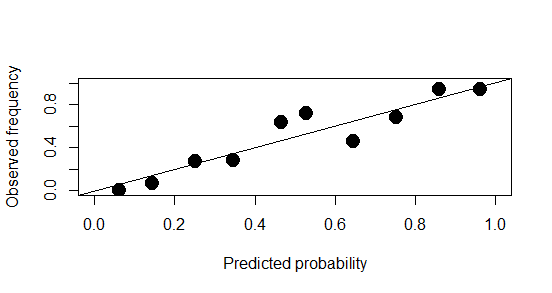


**S4 Appendix.** Calibration plots of the predictive performance of the best latent selection difference model (Table 2) to identify environmental variables that explain habitat selection by wild boar and free-ranging pigs. The observed frequencies of free-ranging pig locations in the validation dataset are plotted as a function of the predicted probability that the habitat would be used by free-ranging pigs in relation to wild boar.
